# Population and ultra-deep sequencing for tropism determination are correlated with Trofile ES: genotypic re-analysis of the A4001078 maraviroc study

**DOI:** 10.1186/1758-2652-13-S4-P128

**Published:** 2010-11-08

**Authors:** S Portsmouth, S Valluri, M Daeumer, B Thiele, H Valdez, M Lewis, C Craig, A Thielen, I James, J Demarest, J Heera

**Affiliations:** 1 Pfizer, Inc, 235 East 42nd Street, New York, USA; 2 Pfizer Inc, New York, USA; 3 Institute of Immunology and Genetics, SEQ.IT GmbH, Kaiserslautern, Germany; 4 Max-Planck-Institute for Informatics, Saarbrücken, Germany; 5 Pfizer Global Research, Sandwich, UK; 6 ViiV Healthcare, Research Triangle Park, USA; 7 Pfizer Global Research, New London, USA

## Background

A4001078 is a study in therapy naive patients of Maraviroc (MVC) plus boosted atazanavir. The Trofile ES (ESTA) was used to determine tropism at Screening. Few re-analyses of genotypic tropism have examined all screened and non-reportable (NR) populations. We aimed to define correlations between methods at screening and evaluate the quantity of X4 using virus in discordant results using ultra-deep sequencing (UDS).

## Methods

Population and UDS methods were employed on 178 of 220 screened subjects and 121 enrolled subjects. Correlation between methods was explored and the quantity of X4-using virus in both discordant and concordant samples was measured using UDS.

## Results

ESTA defined 123 (69%) as R5, 39 (22%) as Dual or Mixed tropism (D/M) and 16 (9%) as NR. Population sequencing (single amplification) defined 146 (82%) as R5, 26 as X4, and 6 tests were non reportable [Either failure to get a PCR product (no result for both, population sequencing and UDS) or non-evaluable Sanger traces]. Correlation between population and UDS for R5 use was 95%. Of the patients screened as R5 by population sequencing, UDS showed a median of 0% X4 with only 3 of 114 results being over 2% X4 use, suggesting this method is suitable for selecting individuals for CCR5 antagonist therapy. All Trofile NR results were reportable by population sequencing and showed tropism results consistent with the overall population.

## Conclusions

Population sequencing appropriately identified patients with <2% CXCR4 using virus and who would be suitable for CCR5 antagonist therapy.

**Figure 1:**
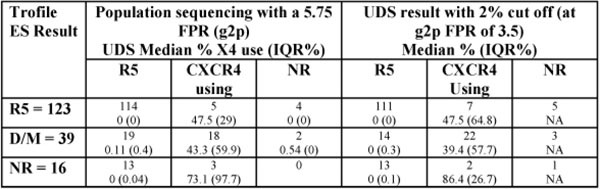
Correlation between methods and quantity of X4 use by UDS in concordant and discordant results and quantity of X4 using virus by UDS.

